# Validating the caregiver self‐efficacy in contribution to self‐care scale Thai version for stroke: A psychometric evaluation

**DOI:** 10.1002/nop2.1990

**Published:** 2023-08-29

**Authors:** Jom Suwanno, Nuntaporn Klinjun, Kannika Srisomthrong, Matthew Kelly, Marzukee Mayeng, Juk Suwanno

**Affiliations:** ^1^ School of Nursing Walailak University Nakhon Si Thammarat Thailand; ^2^ The Excellent Center of Community Health Promotion Walailak University Nakhon Si Thammarat Thailand; ^3^ Faculty of Nursing Prince of Songkla University Songkhla Thailand; ^4^ Department of Global Health, Research School of Population Health Australian National University Canberra Australian Capital Territory Australia; ^5^ Department of Epidemiology, Faculty of Medicine Prince of Songkla University Songkhla Thailand; ^6^ Hatyai Stroke Center Hat Yai Hospital Songkhla Thailand

**Keywords:** caregiver, nursing, self‐efficacy, stroke, validity

## Abstract

**Aim:**

To test the validity and reliability of the Caregiver Self‐Efficacy in Contribution to Self‐Care Scale Thai Version (CC‐Self Efficacy Scale (Thai)) for Stroke.

**Design:**

A cross‐sectional study was undertaken from September to December 2022.

**Methods:**

Four hundred thirty‐four caregivers of people with stroke were selected from the registry of stroke patients in primary care units or hospitals following inclusion criteria. The research assistants collected information when the caregiver took a patient for a doctor's appointment or visited the patient's and caregiver's home.

**Results:**

The 434 caregivers had a mean age of 48 years, female 77.67%, 51.97% child or grandchild of patients, and 72.85% living with the patient. Ten items of the CC‐Self Efficacy Scale (Thai) were normally distributed and appropriate for exploratory factor analysis (EFA). EFA suggested three‐factor model. The confirmatory factor analysis (CFA) of the three‐factor model was an unfit model, with the root mean square error of approximation (RMSEA) = 0.09. We regrouped items based on content to create six‐factor model. CFA supported the six‐factor model of CC‐Self Efficacy Scale (Thai) questionnaire with the reliability judged by McDonald's omega being 0.87. The 434 sample size was enough for EFA and CFA. The CC‐Self Efficacy Scale (Thai) with the six‐factor model is appropriate for evaluating the caregiver confidence of people with stroke.

## 1 INTRODUCTION

Stroke is the second spot as the leading cause of death after ischemic heart disease and is a statistically significant cause of disability worldwide (Hankey, 2013; Kuriakose & Xiao, 2020; Lozano et al., 2012; Naghavi et al., 2017), as reported by World Health Organization (WHO, 2022). Several studies of the mortality rate from stroke in the first year ranged from 5.90% to 34.50%. (Bates et al., 2013; Mar et al., 2015; Novbakht et al., 2020; Nambiar et al., 2022). Strokes affect the economy and considerably burden households (Mapulanga et al., 2014; Rochmah et al., 2021; Zhang et al., 2019). A recent review of the economic burden of stroke in eight countries (Lebanon, Colombia, South Korea, United States, Turkey, Denmark, Sweden and South Africa) found an estimated cost of about 1809.50–325,108.80 US, of which 86.20% were medical expenses, and 13.80% were productivity loss and caregiver costs (Rochmah et al., 2021). Furthermore, healthcare costs for strokes in the community were higher than those in hospitals (Tyagi et al., 2018).

Advancements in medical management systems have reduced hospital stay time, and decreased stroke deaths, leading to more survivors with residual physical disabilities (Tyagi et al., 2018). Healthcare costs decreased for inpatient and emergency departments, while primary care service costs increased (Tyagi et al., 2018; Zhang et al., 2019). Despite advances in medical care, stroke continues to be a leading cause of death and long‐term disability globally. These outcomes are connected to several major stroke risk factors and lack of timely access to stroke units (Caprio & Sorond, 2019; Suwanwela, 2014; Venketasubramanian et al., 2017; Nambiar et al., 2022).

Risk factor control strategies are essential in consistently reducing the mortality rate from stroke and preventing recurrent stroke (Caprio & Sorond, 2019). Stroke caregivers are essential to the long‐term care of those suffering from recurrent stroke and modify patients' risk behaviours (Caprio & Sorond, 2019). Most caregivers are family members (e.g. spouses, children and siblings) or close friends, with caregivers and patients influencing each other (Lobo et al., 2021; Tyagi et al., 2018; Vellone et al., 2021). Caring for post‐stroke at home is complicated and varies according to the pathology of the disease, symptoms of post‐stroke, and the stroke patient's need for care in terms of physical, mental, emotional and social aspects (Hekmatpou et al., 2019; Lobo et al., 2021). Caregiver confidence is one of the critical factors influencing caregiver participation in the care of stroke patients (Vellone et al., 2021). Therefore, a tool is needed to measure the confidence of caregivers caring for patients in the community.

De Maria et al. (2021) developed and tested the Caregiver Self‐Efficacy in Contribution to Patient Self‐Care (CSE‐CSC) Scale. This instrument tested the validity and reliability of caregiver self‐efficacy in multiple chronic conditions (MCCs), with the root mean square error of approximation (RMSEA) = 0.07 and the global reliability index for multidimensional = 0.92. The CDE‐CSC was found suitable for use as a caregiver self‐efficacy test of MCCs in seven centrals and southern Italy regions but has not been used by caregivers for single chronic conditions (De Maria et al., 2021). The original CSE‐CSC scale (English) has been translated into CC‐Self Efficacy Scale (Thai) (https://self‐care‐measures.com/project/caregiver‐contribution‐self‐efficacy‐scale‐thai/). One of the authors was the principal translator using a standard forward and backward translation procedure by seven experts (two forward translators, three synthesizers and two back translators). This instrument has been judged valid by nine experts with kappa coefficients of 1.00.

Caregivers play a statistically significant role in helping patients with self‐care (Vellone et al., 2021). Caregiver self‐efficacy refers to an individual's belief in their ability to provide competent effective care for unwell patients. It is a critical determinant of the quality of caregiver‐patient dyads provided after strokes (Boonsin et al., 2021; Honado et al., 2023; Wang et al, 2021). Hence, it is necessary to evaluate caregivers' self‐efficacy in various settings and for particular chronic illnesses. Stroke provides a key example of chronic illness where disability is often involved and care needed. The instrument's CC‐Self Efficacy Scale (Thai) has been developed, and here, we examine the validity and reliability of this scale measure. The results will get the appropriate instrument for healthcare professionals to further explore the caregiver's self‐efficacy in contributing to self‐care in the post‐stroke, leading to better health outcomes for both the caregiver and the stroke survivor.

## 2 METHODS

### 2.1 Study and sample

The cross‐sectional study was conducted from September to December 2022, including 434 caregivers of people with stroke (237 in Songkhla, 81 in Trang, 47 in Nakon Si Thammarat, 40 in Suratthani and 29 in Phatthalung). We focused on the caregivers of people with stroke whom a medical practitioner had diagnosed with the codes I60‐I64 in the International Statistical Classification of Diseases and Related Health Problems (ICD‐10) from the registry of stroke patients in primary care units or hospitals. Sixteen nurses or public health officers in the primary care unit and hospital selected the 434 caregivers with the following inclusion criteria: over 18 years old, primary caregivers who continuously care for stroke patients in the family, and communicating and reading the Thai language. They directly contacted the caregiver to ensure they understood the objectives, and the participants signed informed consent forms before data were collected. Questionnaires were collected when the caregiver took a patient to a doctor's appointment or the stroke patient's home visit.

### 2.2 Measures and data analysis

The questionnaire consisted of two parts. Part 1: Caregiver demographics included sex, age, education, marital status, employment status, family income, relative with the patient, living with, secondary caregiver, year of caregiver and underlying disease. Part 2: Seven experts translated the CSE‐CSC instrument from the original English (https://self‐care‐measures.com/project/caregiver‐contribution‐self‐efficacy‐scale‐english‐2‐2‐2‐2‐2‐2‐2‐2‐2/) into the Thai language through standard forward and backward translation (https://self‐care‐measures.com/project/caregiver‐contribution‐self‐efficacy‐scale‐thai/). One of the researchers, who had doctoral degrees in nursing and extensive experience in cardiovascular health and scale development, was the principal translator.

The CSE‐CSC instrument includes a 10‐item questionnaire rated on a 5‐point (1–5) Likert scale, with scores ranging from ‘not confident’ (score = 1) to ‘very confident’ (score = 5) (Self‐care Measures, 2017a, 2017b). The construct validity of CSE‐CSC showed model fit as follows chi‐square = 92.08 (df = 33, *p* < 0.001), comparative fit index (CFI) = 0.97, Tucker–Lewis Index (TLI) = 0.96, RMSEA = 0.07, (90% confidence interval = 0.05–0.09 and global reliability index for multidimensional = 0.92) (De Maria et al., 2021).

We performed a confirmatory analysis of the CC‐Self Efficacy Scale (Thai) in caregivers of people with stroke in Thailand. Factor analysis investigated the validity of questions using exploratory factor analysis (EFA) and confirmatory factor analysis (CFA). EFA is used to analyse data to extract the new factor structure and examine the interrelationships among variables (Kim et al., 2016; Williams et al., 2010). There are three steps for the EFA (Shrestha, 2021).

Step 1: Test for sampling adequacy and test assumption of factor analysis: The Kaiser–Meyer–Olkin test (KMO) measured the sampling adequacy for factor analysis. The KMO value ranges from 0 to 1; the criteria for consideration are as follows: 0.80–1.0, meritorious; 0.70–0.79, middling; 0.60–0.69, mediocre; 0.50–0.59, miserable; and below 0.50, unacceptable (Hair et al., 2010).

Bartlett's test of sphericity was used to test the null hypothesis for the identity matrix, as follows;

H_o_: The variables are uncorrelated.

H_1_: The variables are correlated.

A statistically significant level of Bartlett's test (*p* < 0.05) indicated that factor analysis is suitable (Hair et al., 2010).

Step 2: Assess the communality of the variables: Principal components analysis (PCA) is for extracting factors. Communalities are the amount of original variance shared within each variable extracted from a common factor in the analysis, ranging from 0 to 1. If the communality is close to 1, most of the information was extracted, and if it is more than 0.50 extra information is needed to explain (Hair et al., 2010).

Step 3: Choosing the number of factors: Parallel analysis is used for deciding factors to extract or retain, considering plots where the eigenvalues of the FA actual data are higher than plots of the FA simulated data line on the Scree plot (Woods & Edwards, 2007; Revelle, 2020). Factor loadings consider the correlation between the original variable and the factors, ranging from −1 to 1. The sum of squared loadings (SS loading) is used to determine the value of a particular factor, considering the SS loading more statistically significant than 1. Then, the varimax‐rotation component analysis is used to extract the factor loadings to ensure that they are uncorrelated or independent of each other (Revelle, 2020).

Finally, we used the CFA to verify a set's factor structure from EFA compared with the new factor structure by the researcher. The model fit used criteria by a chi‐squared test, model significance (*p* < 0.001 considering reject; comparative fit index (CFI), value above 0.90 indicating good fit; and the root mean square error of approximation (RMSEA), values below 0.08 indicating good fit (Hox, 2021). McDonald's omega estimates the total reliability index for a multidimensional scale (Hair, 2010; Revelle, 2020).

We performed all statistical analyses using R version 4.1.3 (R Core Team, 2022) with the psych package, *lavaan* package and *ltm* package.

## 3 ETHICS STATEMENT

This study was part of a study to test the validity and reliability of the scales measure self‐care for individuals, and caregiver contribution to self‐care in persons with stroke. This was approved by the Human Research Ethics Committee, Walailak University (approval no. WUEC‐22–232‐01); the standards specified in the Declaration of Helsinki were used in this study. All participants were informed of the study's rationale and purpose and signed informed consent at the beginning of the study.

## 4 RESULTS

Table 1 shows the sample consisted of 434 caregivers caring for people with stroke, mean age 48.28 + 13.03 years, primarily female, and had achieved an educational level bachelor's degree or higher degree. Most caregivers lived with the patients and were the patients' children or grandchildren. They had been providing care for an average of 7.35 years and had a secondary caregiver for patients. Most of the caregivers did not have underlying diseases.

**TABLE 1:** Demographic data of 434 caregivers caring for people with stroke.

Variable	Frequency (%)
Sex, female	334 (77.67%)
Age, years^a^	48.28 ± 13.03
Highest education	
Bachelor's degree and higher	114 (26.45%)
Senior high school	78 (18.10%)
Intermediate school	75 (17.40%)
Married/In a relationship	335 (77.73%)
Agriculturist	158 (41.15%)
Family income enough to spend, no savings	212 (49.77%)
Relative with patient, Child/Grandchild	224 (51.97%)
Living with patient	314 (72.85%)
Secondary caregiver	313 (73.65%)
Years of caregiver	7.35 ± 5.80 range (1 month‐30 years)
No underlying disease	288 (66.36%)

^a^ Mean ± Standard deviation.

Table 2 shows the descriptive statistics for individual items of the CC‐Self Efficacy Scale (Thai). All the CC‐Self Efficacy Scale (Thai) items were normally distributed.

**TABLE 2:** Descriptive statistics of individual items in the CC‐Self Efficacy Scale (Thai).

Items of caregiver self‐efficacy in contribution to self‐care scale^a^	*N*	*M*	SD.	Skewness	Kurtosis
In general, in reference to the person you care for, how confident you are that you can					
Keep the illness of the person you care for stable and free of symptoms	434	3.63	0.87	−0.36	−0.11
2 Follow the treatment plan that has been given to the person you care for?	433	3.77	0.85	−0.33	−0.22
3 Persist in following the treatment plan even when difficult?	433	3.80	0.82	−0.56	0.35
4 Routinely monitoring the condition of the person you care for?	434	3.78	0.83	−0.30	−0.21
5 Persist in routinely monitoring the condition of the person you care for even when difficult?	434	3.62	0.81	−0.37	0.22
6 Recognize changes in the health of the person you care for if they occur?	434	3.60	0.85	−0.33	0.78
7 Evaluate the importance of symptoms?	434	3.62	0.88	−0.35	0.07
8 Do something to relieve symptoms of the person you care for?	434	3.49	0.84	−0.05	−0.46
9 Persist in finding a remedy for symptoms of the person you care for even when difficult?	434	3.56	0.90	−0.21	−0.17
10 Evaluate how well a remedy works?	434	3.58	0.84	−0.23	−0.19

^a^ Self‐care Measures, 2017a, 2017b.

### 4.1 Exploratory factor analysis (EFA)

#### 4.1.1 Test for sampling adequacy and test assumption of factor analysis

The KMO measure of sampling adequacy was 0.93 and the Measures of Sampling Adequacy (MSA) for individual variables were 0.89–0.95, meaning the sampling is appropriate for exploratory factor analysis (EFA). Bartlett's test of sphericity was less than 0.001, meaning we can accept the hypothesis that the 10 variables were related to each other for EFA (Table 3).

**TABLE 3:** Kaiser–Meyer–Olkin measure of sampling adequacy and Bartlett's test of sphericity.

Kaiser–Meyer–Olkin measure of sampling adequacy	0.93
Bartlett's test of sphericity approx. chi‐square	3119.34
df	45
Sig	0.000*

* *p* < 0.001.

#### 4.1.2 Assess the communality of the variables

PCA was used in this test. Values of extracted communalities were 0.59–0.69, meaning that more of the variance of individual items was explained (Table 4).

**TABLE 4:** Communalities of variables in the Thai CSE‐CSC.

Items of caregiver self‐efficacy in contribution to self‐care scale*	Initial	Extraction communalities
Item 1	1.000	0.66
Item 2	1.000	0.59
Item 3	1.000	0.59
Item 4	1.000	0.64
Item 5	1.000	0.65
Item 6	1.000	0.63
Item 7	1.000	0.62
Item 8	1.000	0.69
Item 9	1.000	0.66
Item 10	1.000	0.65

* Extraction communalities more than 0.4.

#### 4.1.3 Choosing the number of factors

**FIGURE 1:**
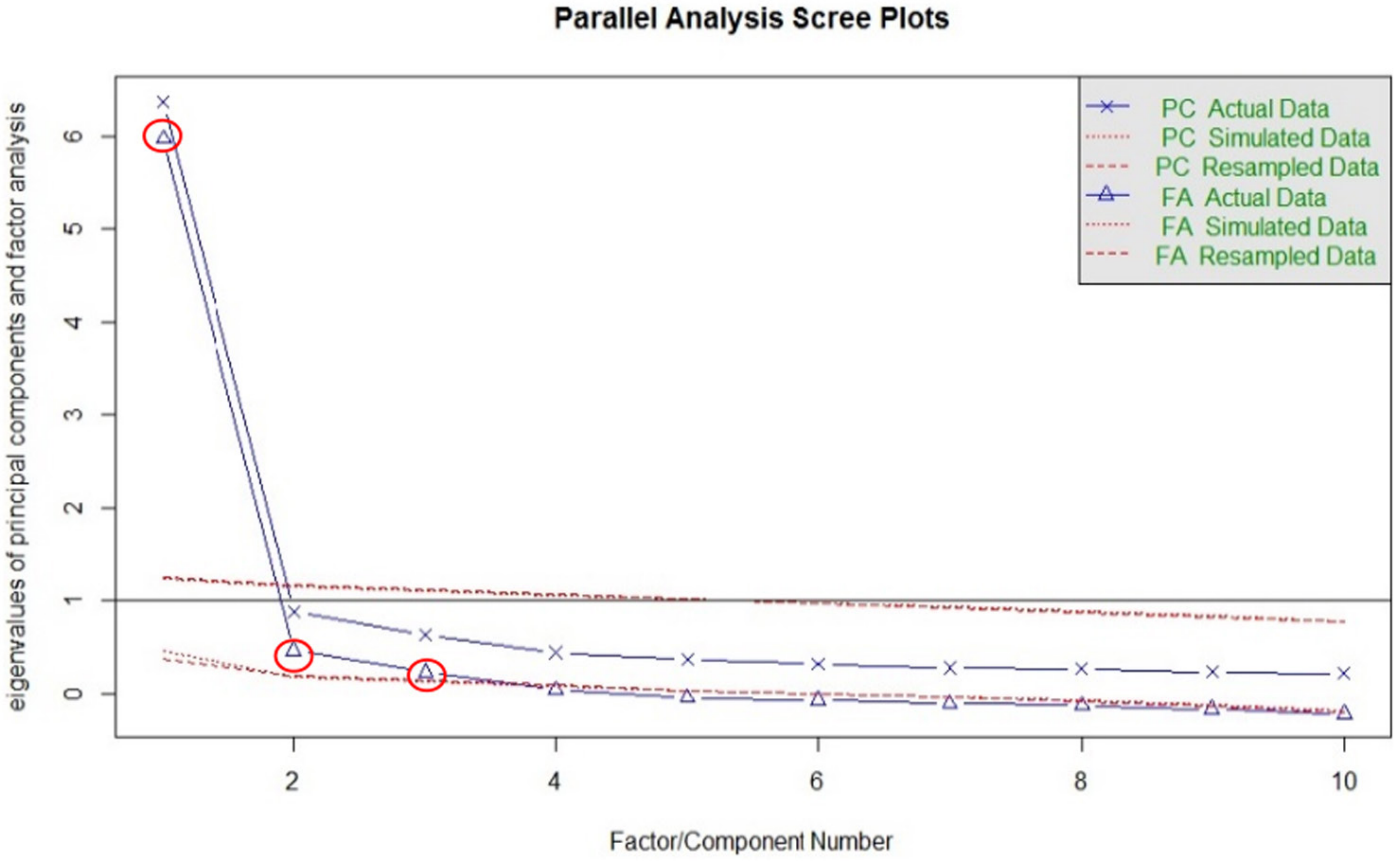
Parallel analysis scree plots, parallel analysis suggests three factors (*R Output*).

Parallel analysis showed the relationship between items' variance and the number of retained items with scree plots. Figure 1 shows three factors considered with a line of FA actual data above FA simulated data, meaning that these three factors were retained. Each scale's eigenvalues were factor 1 = 5.97, factor 2 = 0.46 and factor 3 = 0.23. The sums of squared loading of factor 1, factor 2 and factor 3 were 2.92, 2.27 and 1.82, respectively. Factor 1 consisted of four items (items 7, 8, 9 and 10), and factor loading was 0.69–0.74. Factor 2 consisted of three items (items 1, 2 and 3), and factor loading was 0.63–0.83. Factor 3 consisted of three items (items 4, 5 and 6), and factor loading was 0.55–0.67.

### 4.2 Confirmatory factor analysis (CFA)

The three‐factor model from EFA was analysed for CFA. Three‐factor model was rejected as follow: chi‐square = 146.35 (df = 32, *p* < 0.001), CFI = 0.96, TLI = 0.95, SRMR = 0.04, RMSEA = 0.09 (90% CI = 0.08–0.11).

**FIGURE 2:**
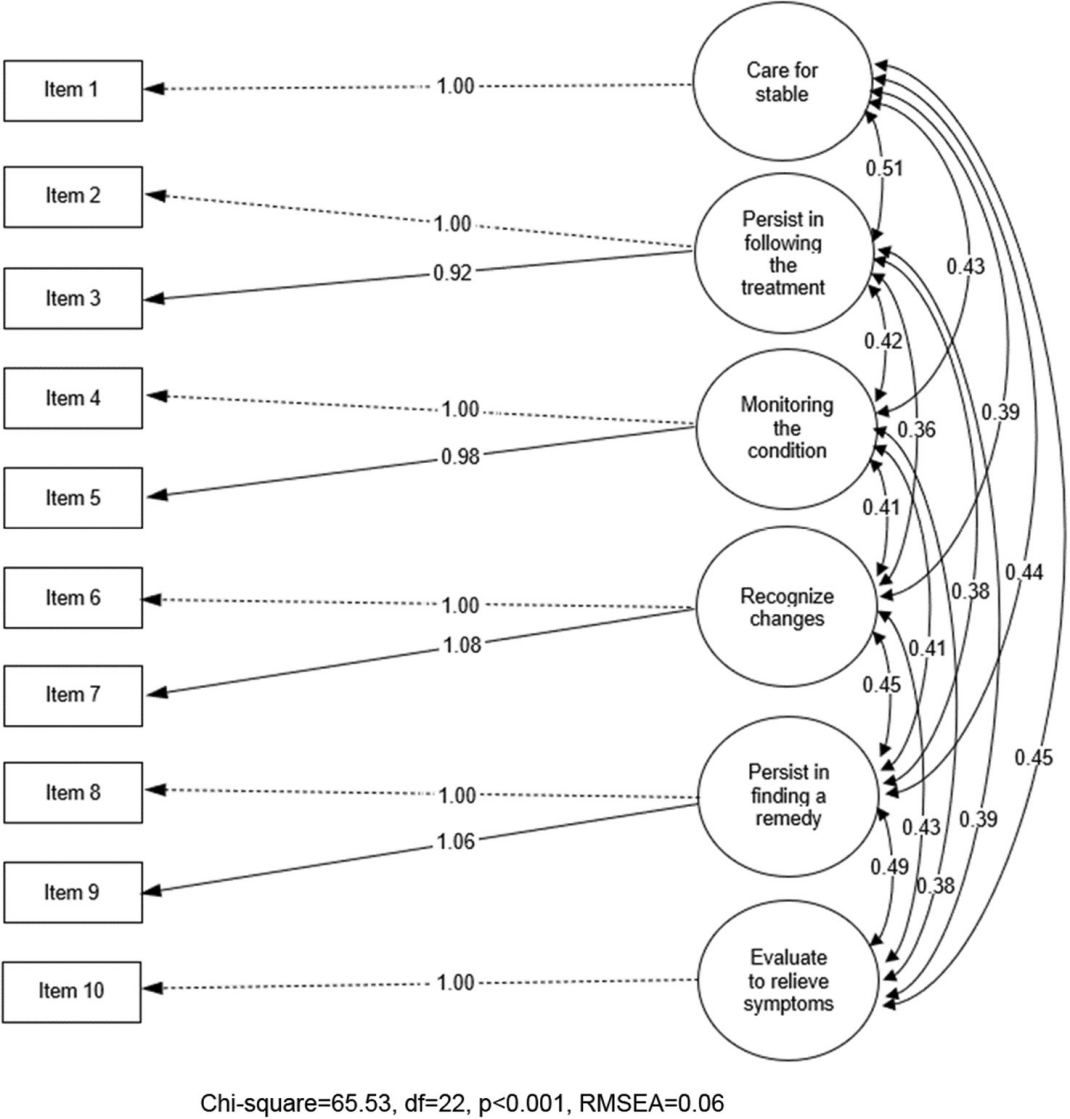
Result of the confirmatory factor analysis (CFA) for the CC‐Self Efficacy Scale (Thai).

We rematched items assessing items and the content to a six‐factor model: item 1, item 2 and item 3, item 4 and item 5, item 6 and item 7, item 8 and item 9, and item 10. Figure 2 shows the factor structure's similarity and renamed six factors: F1 = care for stable; F2 = persist in following the treatment; F3 = monitoring the condition; F4 = recognize changes; F5 = persist in finding a remedy; F6 = evaluate to relieve symptoms. The six‐factor model fit indices were as follows: chi‐square = 65.53 (df = 22, *p* < 0.001), CFI = 0.99, TLI = 0.97, SRMR = 0.02, RMSEA = 0.06 (90% CI = 0.05–0.09) (Figure 2). Tucker–Lewis Index (TLI) was 0.99, meaning that the model was consistent with the empirical data. The reliability index for the multidimensional scale as McDonald's omega was 0.87.

## 5 DISCUSSION

This study aimed to test the psychometric characteristics of the CC‐Self Efficacy Scale (Thai) questionnaire to measure caregiver self‐efficacy in people with stroke. Based on the study, a particularly striking result of Thai caregivers of stroke patients' results was their tendency to be female, married, child/grandchild, bachelor's degree and higher degree, and living with the patient (Boonsin et al., 2021; Muangpaisan et al., 2010; Srisuk et al., 2021).

The present study tested the normal distribution regarding skewness and kurtosis values. The skewness values were found in the range of −0.56 to −0.05, and the kurtosis values were found in the range of −0.46 to 0.35. The most commonly considered values between −1.96 and +1.96 are acceptable normal distributions (Hair et al., 2010). The principal results show that the sample size in this research, 434 caregivers, was excellent for FA with KMO = 0.93, and Bartlett's test of sphericity indices supported the hypothesis with a *p*‐value < 0.001 (Hair et al., 2010). Considering the factor loading, each item of the three‐factor model was more statistically significant than 0.50. Parallel analysis suggested that a three‐factor model was adequate. Then, the variable incorporating the three‐factor model could be analysed in the next step (Hair et al., 2010), and the factor loadings were adequate (>0.30) (Hair, 2010). By comparison, De Maria et al. (2021) study tested the validity of the CSE‐CSC adequate for two‐factor. The EFA three‐factor model was validated to confirm with CEA. CFA is used to test the fit of a hypothesized factor structure or confirm the validity of the factor structure (Mueller & Hancock, 2015). The analysis of the CEA for three‐factor model found that the components were inconsistent with the empirical data, with the *p*‐value of the chi‐squared less than 0.05 and the RMSEA 0.091. The RMSEA values below 0.08 indicate a good fit (Hox, 2021). Therefore, the model modification can be adjusted without affecting the outcome.

To optimize the model of CFA, we consider the content of the CC‐Self Efficacy Scale (Thai). We found four items of the CC‐Self Efficacy Scale (Thai) questionnaire that might have redundant meanings. Hence, the interpretations are not different in Thai as follows, ‘Follow the treatment plan’ and ‘Persist follow the treatment plan’, meaning persist in following the treatment; ‘Routinely monitoring the health condition’ and ‘Persist in routinely monitoring the health condition’ meaning as monitoring the condition; ‘Recognize changes in the health’ and ‘Evaluate the importance of symptoms’ meaning as recognizing changes in the health; ‘Do something to relieve the symptoms’; and ‘Persist a remedy for symptoms’ meaning as persist in finding a remedy. Therefore, we matched the redundant meaning in the six‐factor model, indicating care for caring for stable, persist in following the treatment, monitoring the condition, recognizing changes in the health, persist in finding a remedy and evaluate to relief systems. Cultural factors in Thai communication might be the reason for this. The six‐factor model's RMSEA value of 0.06 in this sample indicates an acceptance model inconsistent with De Maria et al. (2021) two‐factor study. One indicated self‐efficacy in self‐care maintenance and monitoring, and the other indicated self‐care management (De Maria et al., 2021). The 434 caregivers in this study were accurate estimates of CFA, of which 200 had a reasonable sample size (Hox, 2021). However, the chi‐squared value of the six‐factor model was found to be statistically significant (*p*‐value = 0.000) means rejecting the model. The large sample size makes the test statistically significant (Hox, 2021).

Reliability for the multidimensional scale was adequate for the CC‐Self Efficacy Scale (Thai) questionnaire, with 0.87. This means that the CC‐Self Efficacy Scale (Thai) questionnaire was suitable for measuring Thai caregiver self‐efficacy in people with stroke. This reliability index for the multidimensional scale of the CC‐Self Efficacy Scale (Thai) of Stroke is close to the CSE‐CSC of multiple chronic conditions (MCCs) with 0.92 (De Maria et al., 2021).

The limitation of this study was that it only studied a stroke caregiver's self‐efficacy in caring for stroke patients; therefore, a study of the actual outcomes of caring for stroke patients should be considered. Other limitations are that the caregiver's self‐efficacy test only examines one part of caring for stroke patients. Therefore, a study of the other instruments should be considered further, such as the caregiver contribution to self‐care of stroke, the ENRICHD social support inventory (Vaglio et al., 2004), the brief illness perception questionnaire (Broadbent et al., 2006) and health‐related quality of life measure (Pattanaphesaj, 2014).

## 6 CONCLUSIONS

A validation study of the CC‐Self Efficacy Scale (Thai) questionnaire was conducted. A sample dataset obtained from 434 caregivers of people with stroke showed excellently suitable for EFA and CFA. The CC‐Self Efficacy Scale (Thai) questionnaire gives evidence of construct validity and internal reliability to be applied to caregivers of people with stroke. We recommend using the CC‐Self Efficacy Scale (Thai) for sustainability in caregiver evaluations in community health nursing.

## AUTHOR CONTRIBUTIONS

JS contributed to the conceptualization, methodology, validation, data collection, funding acquisition and writing—original draft, and approved the final version of the manuscript. NK contributed to the conceptualization, methodology, validation, investigation, data collection, analysis, funding acquisition, w—original draft and supervision, reviewed the results, and approved the final version of the manuscript. KS contributed to the methodology, validation, investigation and data collection, and approved the final version of the manuscript. MM served as the statistician, contributed to the analysis, data curation and interpretation of results, reviewed the results, and approved the final version of the manuscript. MK served as the English Editor, contributed to the conceptualization and writing—review editing, and reviewed and approved the final version of the manuscript. JS contributed to the validation and data collection, and approved the final version of the manuscript.

## FUNDING INFORMATION

This work was supported by Walailak University, Thailand, an individual scholarship [grant number WU‐IRG‐65‐028]. The funder has no role in the study design, data collection and data analysis or as a policy maker.

## CONFLICT OF INTEREST STATEMENT

The authors declare no conflict of interest. The funders had no role in the design of the study; in the collection, analyses or interpretation of data; in the writing of the manuscript; or in the decision to publish the results.

## Data Availability

The data that support the findings of this study are available from the corresponding author upon reasonable request.
